# An open dataset for human SSVEPs in the frequency range of 1-60 Hz

**DOI:** 10.1038/s41597-024-03023-7

**Published:** 2024-02-13

**Authors:** Meng Gu, Weihua Pei, Xiaorong Gao, Yijun Wang

**Affiliations:** 1 Key Laboratory of Solid-State Optoelectronics Information Technology, Institute of Semiconductors, Chinese Academy of Sciences, Beijing, 100083 China; 2 College of Materials Science and Opto-Electronic Technology, University of Chinese Academy of Sciences, Beijing, 100049 China; 3 School of Future Technology, University of Chinese Academy of Sciences, Beijing, 100049 China; 4 Department of Biomedical Engineering, School of Medicine, Tsinghua University, Beijing, 100084 China; 5 Chinese Institute for Brain Research, Beijing, 102206 China

**Keywords:** Biomedical engineering, Neuroscience

## Abstract

A steady-state visual evoked potential (SSVEP)-based brain-computer interface (BCI) system relies on the photic driving response to effectively elicit characteristic electroencephalogram (EEG) signals. However, traditional visual stimuli mainly adopt high-contrast black-and-white flickering stimulations, which are easy to cause visual fatigue. This paper presents an SSVEP dataset acquired at a wide frequency range from 1 to 60 Hz with an interval of 1 Hz using flickering stimuli under two different modulation depths. This dataset contains 64-channel EEG data from 30 healthy subjects when they fixated on a single flickering stimulus. The stimulus was rendered on an LCD display with a refresh rate of 240 Hz. Initially, the dataset was rigorously validated through comprehensive data analysis to investigate SSVEP responses and user experiences. Subsequently, BCI performance was evaluated through offline simulations of frequency-coded and phase-coded BCI paradigms. This dataset provides comprehensive and high-quality data for studying and developing SSVEP-based BCI systems.

## Background & Summary

Brain-computer interfaces (BCIs) provide a new medium for communication between humans and computers, which can convert signals of brain activities into commands to control external devices without the participation of peripheral nerves and muscles^1–3^. Among many BCI paradigms, the steady-state visual evoked potential (SSVEP)^4^-based BCI has attracted widespread attention because of its non-invasiveness, high signal-to-noise ratio (SNR) and high information transfer rate (ITR)^5,6^.

SSVEPs are the sustained cortical responses to periodic visual stimuli. SSVEP has frequency following characteristics, that is, it contains a series of frequency components that are integer multiples of the stimulation frequency, of which the fundamental frequency and double frequency components are the most significant^7^. Depending on the range of the stimulation frequency, SSVEP responses can generally be divided into three bands: low frequency band (less than 12 Hz), medium frequency band (12–30 Hz), and high frequency band (higher than 30 Hz)^7,8^.

Different stimulation frequencies elicit different SSVEP amplitudes, which affect the system performance of SSVEP-BCIs. Historically, research on the relationship between stimulus frequency and response amplitude adopted light emitting diodes^9^ (LED) as visual stimulators^10^. For example, Herrmann *et al*. used a flickering light with a stimulus frequency of 1–100 Hz (with an interval of 1 Hz) to measure SSVEPs, and the results showed that the highest amplitude of SSVEP appeared at 10 Hz, and local peaks appeared around 20 Hz, 40 Hz, and 80 Hz^11^. Pastor *et al*. used strobe lamps to generate a total of 14 sampling frequencies in the range of 5–60 Hz, and found that the occipital region of the brain responded most strongly to the stimulation of 15 Hz, and the brain regions activated were different at different stimulation frequencies^12^. Ferreira *et al*. explored the SSVEP in the frequency range of 5.5–86 Hz using green LED emitters and found that the peak of fundamental frequency response appeared at 10 Hz, 16 Hz, and 31 Hz, respectively^13^.

With the continuous development of computer display technology in recent years, the use of liquid crystal display (LCD) monitors can change stimulation parameters (such as size, shape, etc.) more flexibly, resulting in an upward trend of the SSVEP-BCIs based on the refresh rate of the display^14^. Nakanishi *et al*. constructed a 40-target SSVEP-BCI speller with a frequency range of 8–15.8 Hz (interval 0.2 Hz) and obtained an ITR of up to 325.33 bits/min^15^. Chen *et al*. built an online high frequency (31–34.5 Hz, with an interval of 0.25 Hz) SSVEP-BCI system with 16 targets, which reached an average ITR of 153.79 bits/min^16^. Jiang *et al*. developed a four-target phase-coded SSVEP-BCI system with a frequency of 60 Hz based on an LCD monitor with a 240 Hz refresh rate, which took advantage of the characteristics of low flickering perception of high-frequency stimuli but obtained a low ITR (29.8 bits/min) due to weak response^17^. Compared with high-frequency visual stimuli, the low-frequency and medium-frequency visual stimuli can evoke more robust responses but are easier to cause visual fatigue.

In the design of high-performance and user-friendly SSVEP-BCI systems, in addition to the stimulus frequency, stimulus luminance^18^ is also an important parameter. Rahimi-Nasrabadi *et al*. showed that the sensitivity of light and dark contrast in the visual cortex is affected by changes in luminance^19^. Duszyk *et al*. found that the primary visual cortex is more sensitive to high luminance stimuli, but the reduction of stimulus luminance will effectively improve the user experience^20^. Ladouce *et al*. also demonstrated the effectiveness of lowering the stimulation amplitude depth in the 13–24 Hz frequency band to improve user experience^21^, and achieved high classification performance using the task-related component analysis^15^ (TRCA) algorithm. Ming *et al*. built a 40-target speller at 8–15.8 Hz (interval 0.2 Hz) frequency band using a grid stimulus with a spatial contrast ratio of 50% and obtained an online ITR of 57 bits/min using the unsupervised canonical correlation analysis^22^ (CCA) algorithm. Compared with the traditional high-luminance (i.e., black and white) flickering stimulation, this low-contrast stimulation paradigm achieved comparable performance and improved user experience^23^.

At present, only a few studies have investigated the relationship between SSVEP and stimulus when using computer monitor-based visual stimulators. The characteristics of different modulation depths (the ratio between the maximum and minimum luminance) as a function of stimulus frequency remain unknown. Due to the different methods and devices of stimulus presentation, it is difficult to reveal the common effect of stimulus luminance. To develop an efficient and comfortable BCI system, choosing an appropriate stimulation frequency and stimulus luminance is essential.

In recent years, the establishment of public SSVEP database is of great significance for paradigm design and algorithm optimization in the SSVEP-BCI field. Wang *et al*. provided a 40-target SSVEP benchmark dataset involving a total of 35 subjects, which used joint frequency and phase coding in the frequency range of 8–15.8 Hz (interval 0.2 Hz)^14^. Lee *et al*. provided a BCI dataset comprising 54 participants with three primary paradigms (i.e., motor imagery (MI), event-related potential (ERP), and SSVEP) and conducted a comprehensive comparison^24^. Lee *et al*. also collected a mobile BCI dataset comprising 24 participants and evaluated the quality of SSVEP signals in various motion environments^25^. Choi *et al*. presented a multi-day and multi-band SSVEP dataset based on measurements from 30 participants, which spanned three different frequency bands (low: 5.0, 5.5, 6.0, and 6.5 Hz; medium: 21.0, 21.5, 22.0, and 22.5 Hz; high: 40.0, 40.5, 41.0, and 41.5 Hz) during 2 days^26^. Zhu *et al*. provided an open dataset based on a wearable SSVEP-BCI system, and this dataset consisted of 8-channel EEG data from 102 subjects performing a 12-target (frequencies spanning 9.25–14.75 Hz) SSVEP-BCI task^27^. Liu *et al*. published a benchmark database for BCI application (BETA), which contained 64-channel EEG data from 70 subjects performing a 40-target (frequencies spanning 8–15.8 Hz) spelling task^28^. Furthermore, Liu *et al*. developed a 9-target eldercare-oriented benchmark database of SSVEP-BCI for the aging population (eldBETA). This dataset involved 100 subjects, and the stimulus frequency was set to 8–12 Hz (interval 0.5 Hz)^29^. However, an open SSVEP dataset with higher frequency resolution over a wide frequency range for classical stimulus paradigms is still missing for the field. Especially for the unpopular ultra-low frequency band and ultra-high frequency band, there is still a lot of room for exploration in the optimization of the stimulation paradigm.

This study presents an SSVEP open dataset with a wide frequency range of 1–60 Hz (interval 1 Hz). We systematically explored the influence of stimulus frequency and modulation depth on SSVEP response and user experience. Two different modulation depths were designed for comparison. The dataset contains 64 channels of EEG signals from 30 subjects. Data analysis on SSVEP characters and user experience illustrated the impact of frequency on SSVEP response and user experience under two different modulation depths. Offline simulation analysis further demonstrated the efficacy of the dataset for developing SSVEP-BCIs. This dataset aims to provide a comprehensive and high-quality database for studying and developing user-friendly SSVEP-BCI systems.

## Methods

### Participants

A total of 30 healthy subjects (16 females) were recruited for the experiment, ranging in age from 21 to 35 years, with a mean age of 26.5 years. All participants had normal or corrected-to-normal vision. Before the experiment, the participants were instructed to be familiar with the experimental protocol, stated that they were aware of their right to withdraw at any point during the process, and agreed to open publication of their data after the experiment was completed. Every participant signed an informed consent form approved by the institution review board of Tsinghua University (NO. 20230058).

### Stimulus Presentation

**Fig. 1:**
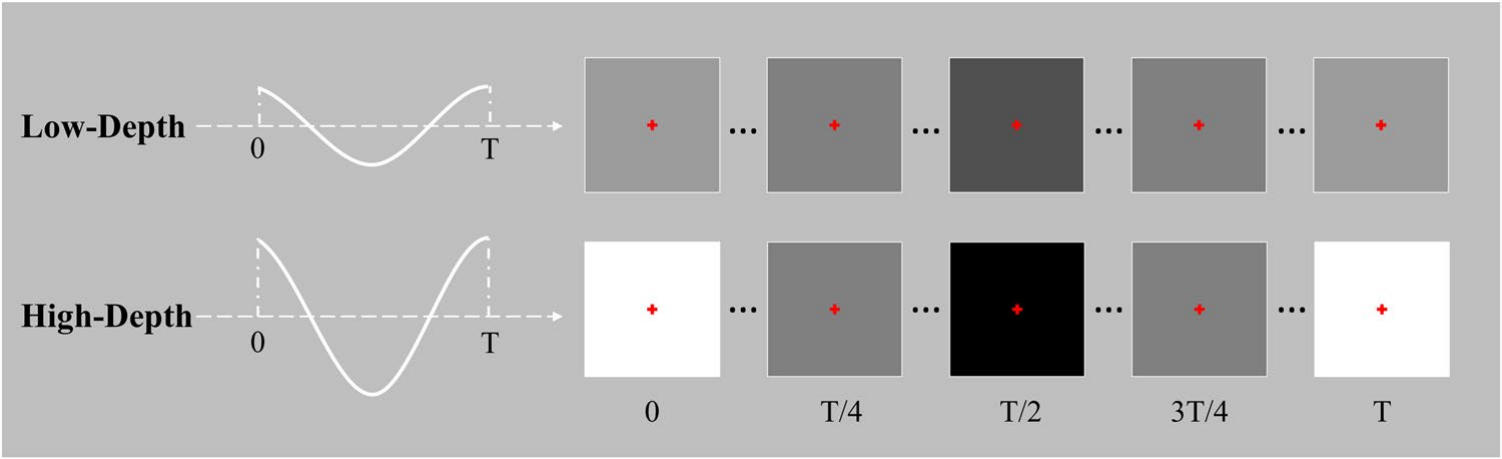
Visual stimuli with two different modulation depths.

Figure 1 shows two flickering stimuli with different modulation depths used in the experiment, where the stimulus with the low modulation depth is denoted as Low-Depth (luminance: 26.31–78.64 cd/m^2^; grayscale value: 0.315–0.613) and the stimulus with the high modulation depth is denoted as High-Depth (luminance: 0.2–207.3 cd/m^2^; grayscale value: 0-1). According to Ming *et al*.’s description of the spatial contrast of the ON-OFF grid stimulus^23^, the flicker luminance range of the Low-Depth stimulus is the same as that of ON-OFF grid stimulus with a Weber contrast ratio of 50%, and the flicker luminance range of the High-Depth stimulus is the same as that of ON-OFF grid stimulus with a Weber contrast ratio of 300% (background luminance: 52.3 cd/m^2^; background grayscale value: 0.5). Weber contrast is calculated as follows:^30^

1 $$\begin{array}{c}{C}_{w}=\frac{{L}_{c}-{L}_{b}}{{L}_{b}}\end{array}$$

where *L*_*c*_ is the highest or the lowest luminance, and *L*_*b*_ is background luminance.

The experiment used a 24.5-inch LCD monitor (Alienware AW2521H) with a screen refresh rate of 240 Hz and a resolution of 1920 × 1080 pixels to present stimulation. Each flickering stimulus was represented by a sine wave inside a 310 × 310 pixels square, and the viewing angle of the entire stimulus was 7.10°. To make the participant better concentrate during the stimulation, a 0.55° red cross fixation point was added in the centre of the stimulation pattern. The stimulus frequency was set to 1, 2, 3, 4… 60 Hz with a 1 Hz interval.

The sampled sinusoidal stimulation method^31^ was implemented by the Psychophysics Toolbox Version 3 (PTB-3) toolbox^32^ under MATLAB R2015b. The grayscale value of the stimulus sequence is calculated as follows:

2 $$\begin{array}{c}Low-Depth:s(f,i)=0.149\,sin\left[2,\pi ,f,(\frac{i}{fr}),+,\frac{\pi }{2}\right]+0.464\end{array}$$

3 $$\begin{array}{c}High-Depth:s(f,i)=0.5\,sin\left[2,\pi ,f,(\frac{i}{fr}),+,\frac{\pi }{2}\right]+0.5\end{array}$$

where fr represents the refresh rate of the screen, i represents the frame index of the sequence, and f indicates the stimulus frequency.

### Experimental Design

**Fig. 2:**
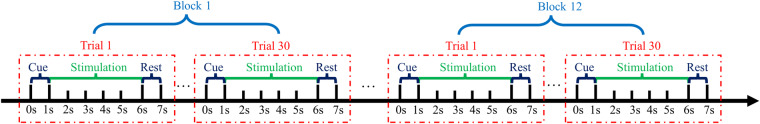
The workflow in each session for each participant.

The experiment included a total of 120 different stimuli (60 stimulation frequencies × 2 modulation depths). During the experiment, the participant sat in a chair in a bright room at a viewing distance of 70 cm from the monitor. Due to the large amount of stimulation patterns, the experiment was separated into four sessions on different days for each participant, and the 15 stimulus frequencies (with an interval of 4 Hz) in each session were listed in Table 1. Each participant completed four sessions in the order specified in Table 1. For each session, the workflow is shown in Fig. 2, with 12 blocks, each containing 30 trials, corresponding to 30 different stimulus patterns (i.e., 15 frequencies × 2 modulation depths). In each block, 30 distinct stimulation conditions randomly appeared. This allowed for the repeated collection of each stimulation condition 12 times. The total duration of each trial was 7 s. In the first second, a red cross at the target centre appeared to remind the participant to focus their eyes on the target center^33^. The stimulus then began to flash for 5 s, during which the participant was required to focus on the target and avoid blinking. The red cross disappeared after the flickering stimulus stopped, and the participant took a short break for 1 s. The selected stimulus duration aimed to meet the requirements for offline simulation of the phase-coding BCI. After the completion of each block, participants had a rest for a certain period (1–10 minutes) to avoid visual fatigue.

**Table 1:** Stimulus frequencies in each session.

Session	Stimulus frequency
1	1, 5, 9, …, 57 Hz
2	2, 6, 10, …, 58 Hz
3	3, 7, 11, …, 59 Hz
4	4, 8, 12, …, 60 Hz

After collecting all sessions of EEG data, participants were asked to score the user experience of each stimulus pattern. Due to the large number of stimulus patterns, a Samsung LC49G95T display monitor (resolution of 5120 × 1440 pixels and refresh rate of 240 Hz) was used to simultaneously present 60 visual stimuli, which were arranged in random order, on the screen. The questionnaire assessment included the following three dimensions: (a) Score each stimulus pattern according to comfort level based on a five-point scale (scales 1–5 correspond to very uncomfortable, uncomfortable, slightly uncomfortable, comfortable, very comfortable, respectively), (b) Score each stimulus pattern according to flicker perception based on a five-point scale (scales 1–5 correspond to very annoying, annoying, slightly annoying, perceptible, imperceptible, respectively), and (c) Score each stimulus pattern according to preference (scales 1–5 correspond to very disgusting, disgusting, neutral, likeable, very likeable, respectively).

### Data Acquisition

During the experiment, a Neuroscan Synamps2 system was used to record 64 channels of EEG data at a sampling rate of 1000 Hz (according to the international 10/20 system), with the reference electrode at the vertex and the electrode impedance always kept below 20 kΩ. Two computers were used for EEG acquisition and stimulus presentation, respectively. The stimulator generated event triggers (stimulus onset) and sent them to the amplifier through a parallel port. Finally, EEG data and synchronous trigger signals were recorded and saved for offline analysis. A built-in 50 Hz notch filter was used to eliminate power line noise, and the band-pass filter was set from 0.1 Hz to 100 Hz to preserve wideband spectral characteristics. These filtering procedures were already incorporated during the data acquisition process.

### Data Preprocessing

The recorded data were pre-processed to facilitate the subsequent data analysis. Continuous EEG data were extracted as stimulus-related epochs, including 5 s responses of SSVEP from the stimulus onset and 0.14 s recordings after the stimulus offset. 0.14 s was set according to the latency delay of the visual system^14^. All data epochs were downsampled from 1000 Hz to 250 Hz.

### Performance evaluation

#### Signal-to-noise ratio (SNR)

The SNR of SSVEP is defined as the ratio of the amplitude of SSVEP at the stimulus frequency to the average amplitude of background EEG activities in adjacent frequency bands. The SNR of SSVEP (in decibels, dB) is calculated as follows:

4 $$\begin{array}{c}SNR=20lo{g}_{10}\frac{Ky\left(f\right)}{{\sum }_{k=1}^{\frac{K}{2}}\left[y\left(f-k\Delta f\right)+y\left(f+k\Delta f\right)\right]}\end{array}$$

where *y*(*f*) is the amplitude at the stimulus frequency *f*, Δ*f* is the frequency resolution of the amplitude spectrum, and *K* is the number of adjacent frequencies that are included in the noise. In this study, Δ*f* is 0.2 Hz, and *K* is set to 8.

#### Information transfer rate (ITR)

ITR is a key indicator widely used to evaluate BCI performance, which is related to the number of targets (*M*), the average target selection time (*T* in seconds), and the classification accuracy (*P*). The definition of ITR (in bits per min, bpm) is as follows:^2^

5 $$\begin{array}{c}ITR=\left[{log}_{2}M+P{log}_{2}P+\left(1-P\right){log}_{2}\frac{1-P}{M-1}\right]\times \left(\frac{60}{{T}}\right)\end{array}$$

## Data Records

The dataset is freely available from the Figshare^34^. The experimental records include EEG data from 30 subjects indexed as s1-s30, questionnaire results on subjective feelings, and experiment information related to the dataset. Continuous EEG data are stored in the EEG Brain Imaging Data Structure (BIDS^35^) format, and for each participant, segmented epoch data are provided in MATLAB MAT format. Questionnaire results are stored in both MATLAB MAT and CSV file formats for convenient downloading and usage. Additional information about the experiment, including participant details and electrode channel information, is separately stored in CSV file format within the repository^34^.

### EEG data

Two types of EEG data are provided: continuous EEG recordings and epochs of raw data that have been segmented based on stimulus labels.

**Fig. 3:**
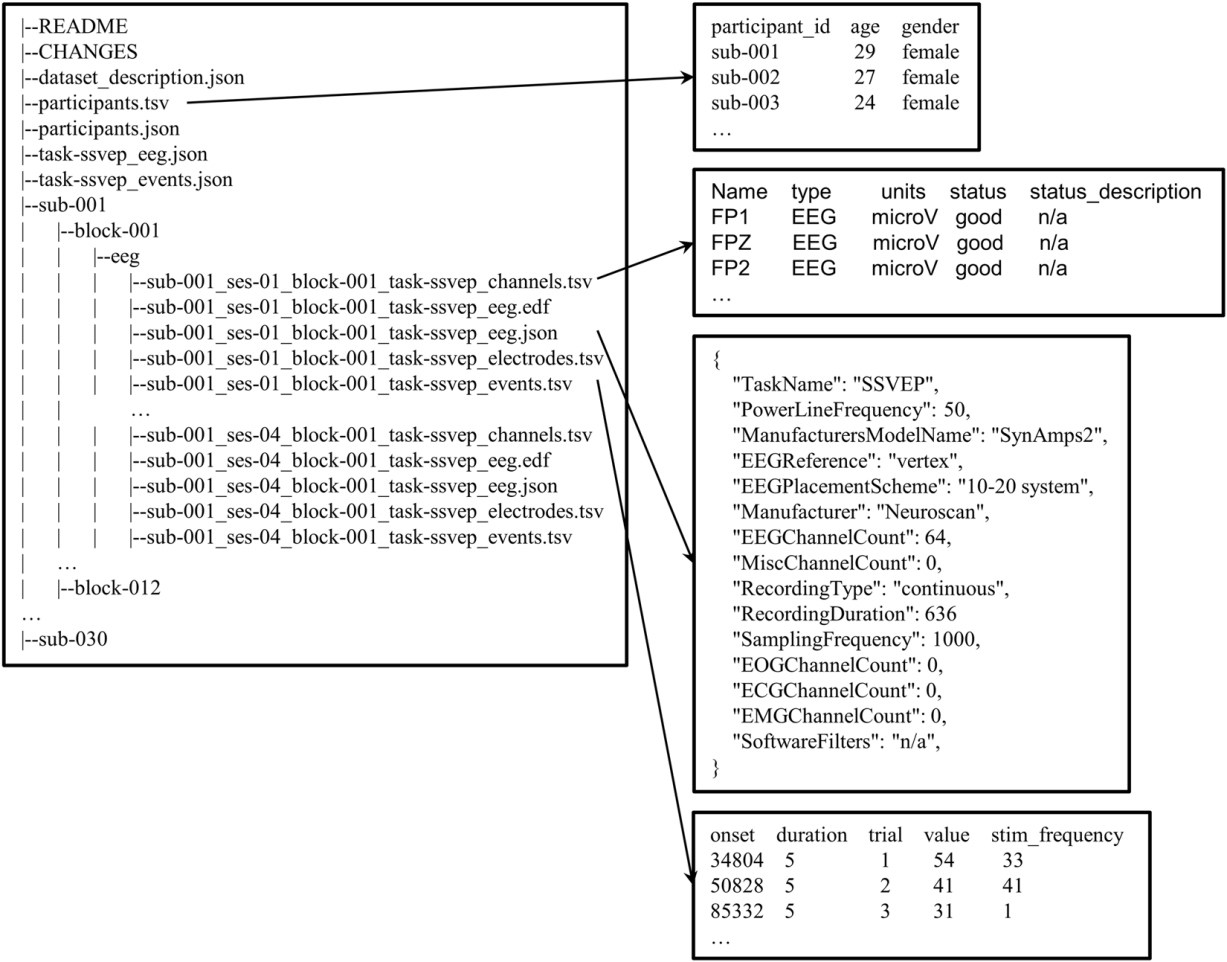
A preview of raw EEG data recordings of the dataset^34^. The data records were created according to the EEG-BIDS^35^. The raw EEG were stored in the European data format (“.edf”). The prefix “sub” denotes the participant and “ses” denotes the session.

Following the EEG-BIDS^35^ standard, the raw EEG data for each participant are organized within a designated folder (e.g., “sub-001”). The EEG data are stored in “.edf” file, while the “.tsv” and “.json” files contain relevant information about the experiment. A preview of the data structure can be seen in Fig. 3.

Another type of segmented epoch data related to stimuli is captured, with each participant’s data forming an independent MATLAB MAT file. Each file is named following the convention “data_Subject index_Number of channels” (e.g., data_s1_64.mat, data_s2_64.mat,…, data_s30_64.mat). For each subject, the data are structured as a 5-dimensional matrix (condition × channel × time point × frequency × block). Specifically, the condition dimension has a size of 2, corresponding to two distinct modulation depths (Low-Depth and High-Depth). The number of channels is 64, and the names and locations of these channels can be found in the experiment information files. The time point duration is 5.14 s, representing a stimulation time of 5 s and a post-stimulation duration of 0.14 s. The stimulus frequency dimension consists of 60 levels, corresponding to stimulation frequencies ranging from 1 to 60 Hz. There are 12 blocks, indicating 12 repeated stimuli for each stimulus pattern.

### Questionnaire scoring

The results of the user experience questionnaires completed by all participants are stored in a MATLAB MAT file and a CSV file, both named “Sub_score.” In the MATLAB MAT file, the scores for all participants are stored in the form of a 4-dimensional matrix (subject × condition × frequency × evaluation dimension). Specifically, there are 30 participants, 2 conditions (corresponding to two distinct modulation depths), 60 stimulus frequencies (ranging from 1 to 60 Hz), and 3 evaluation dimensions (representing comfort level, flicker perception, and preference). In the CSV file, each column represents a participant’s scores for 60 stimulus frequency conditions under a specific evaluation dimension. The rows correspond to the stimulus frequencies, ranging from 1 to 60 Hz, with a step increment of 1 Hz from top to bottom. The column vector names represent specific experimental conditions, where “Sub1_Low_Depth _Comfort_level” indicates the comfort level rating of the first participant for Low-Depth stimuli.

### Experiment information

Participant information is stored in a “Participants_information.csv” file, which specifically includes the label, gender, and age of each participant. The names and locations of the electrode channels can be found in the “Electrode_channels_information.csv” file. This file contains the name, index, angle in polar coordinates, and radius in polar coordinates for each channel. The two files are stored in the repository^34^ together with the dataset.

## Technical Validation

### Stimulus and SSVEP Signal characteristic analysis

**Fig. 4:**
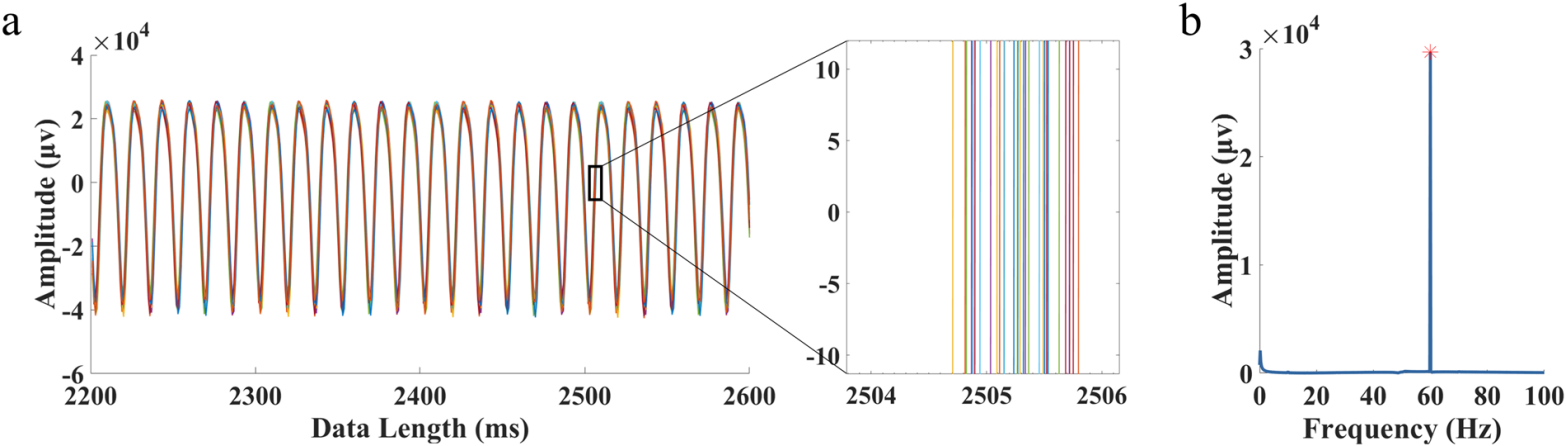
Stimulus signals recorded with a photodiode at a stimulus frequency of 60 Hz. (**a**) Superimposed waveforms of 30 trials with data segments ranging from 2.2 to 2.6 s, including enlarged data segments. (**b**) Amplitude spectrum of stimulus waveforms recorded by the photodiode.

Before the experiment, the refresh rate of the monitor was tested using a photodiode to ensure the stability of the stimulus presentation^36^. In Fig. 4(a), the waveforms of the 60 Hz stimulus signal recorded in 30 trials are depicted. The mean temporal deviation within one block of 30 trials remained 1.1 ms, as shown in the magnified view. Figure 4(b) illustrates that the peak response signal aligns with the stimulus frequency. These results demonstrate the stability of stimulus presentation using a 240 Hz refresh rate.

The subsequent comprehensive analysis of temporal, spectral, and spatial features of SSVEPs validated the reliability of the EEG recordings. EEG topographic analysis employed 64 channels, while the remaining analysis focused on only 9 channels (Pz, PO3/4, PO5/6, POz, Oz, and O1/2) located in the parietal and occipital regions.

**Fig. 5:**
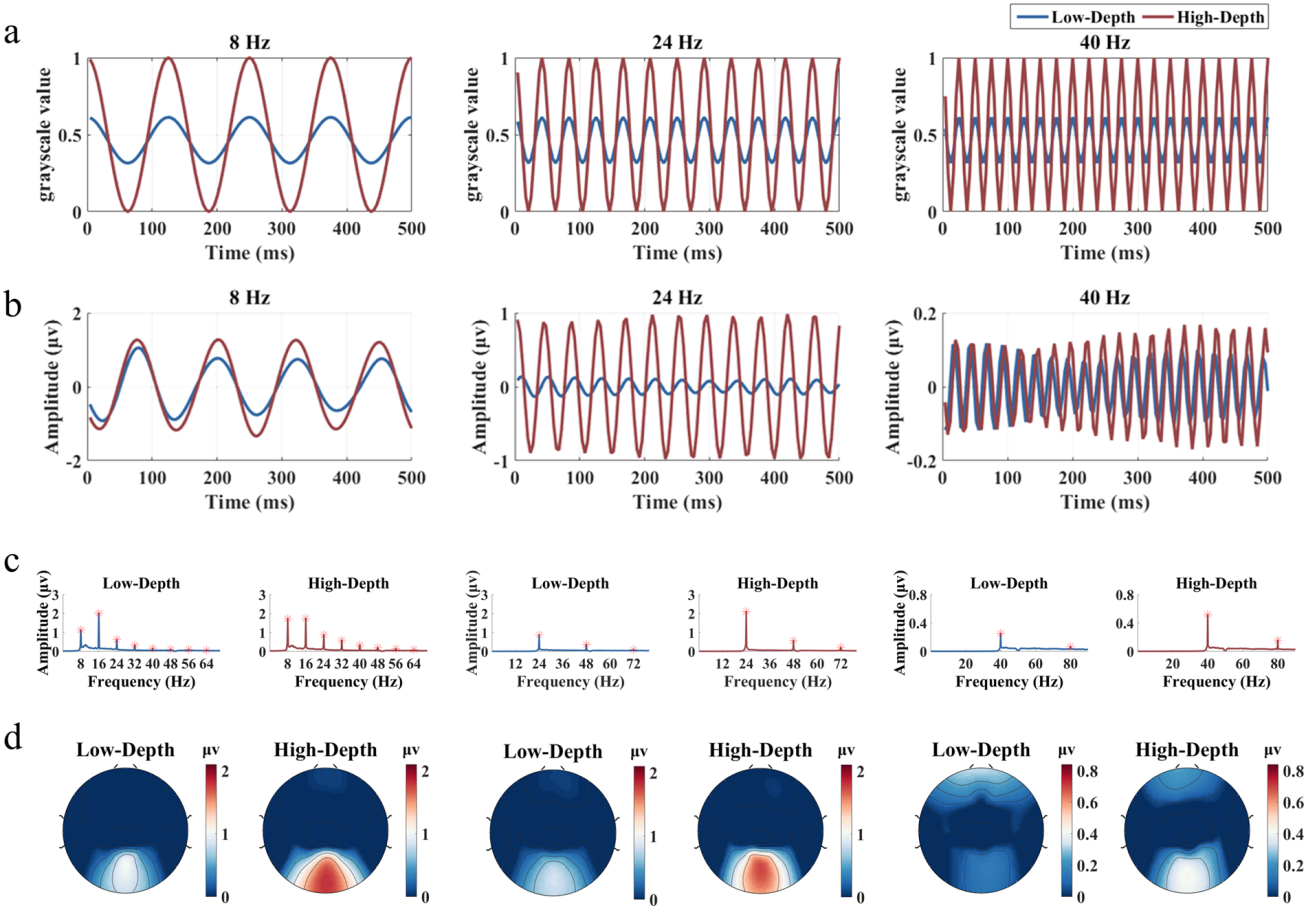
Stimulus signals and the corresponding average SSVEP signals at 8 Hz, 24 Hz, and 40 Hz across all subjects. (**a**) Sampled sinusoidal stimulation signals. (**b**) Averaged SSVEP signals across 9 channels with a duration of 0.5 s. (**c**) Averaged SSVEP amplitude spectra across 9 channels. (**d**) Topographic maps on the amplitude of SSVEPs at the fundamental frequency for all channels on the scalp. The Chebyshev Type I band-pass filters in (**b**) and (**d**) were configured with the parameters [f-0.2, f + 5] Hz, while the band-pass filters in (**c**) were set to [f-0.2, 90] Hz to preserve harmonic responses, where f represents the stimulus frequency.

Figure 5 illustrates the waveforms of the stimulation signals and the characteristics of averaged SSVEP responses at example frequencies (low frequency: 8 Hz, medium frequency: 24 Hz, and high frequency: 40 Hz) across 30 subjects. In Fig. 5(b), the periodicity of SSVEPs is pronounced at these three frequencies. The response amplitude of the Low-Depth stimulus is lower than that of the High-Depth stimulus. Figure 5(c) reveals distinct peaks not only at the fundamental frequency but also at harmonic frequencies. Notably, for the response to the Low-Depth stimulus at 8 Hz, the amplitude of the second harmonic is higher than that of the fundamental frequency. This phenomenon is commonly observed in responses to stimuli in the low frequency band. In Fig. 5(d), areas with strong SSVEP responses are predominantly concentrated in the occipital region of the brain. These findings highlight robust and reliable temporal, spectral, and spatial characteristics for the SSVEP recordings in the dataset.

**Fig. 6:**
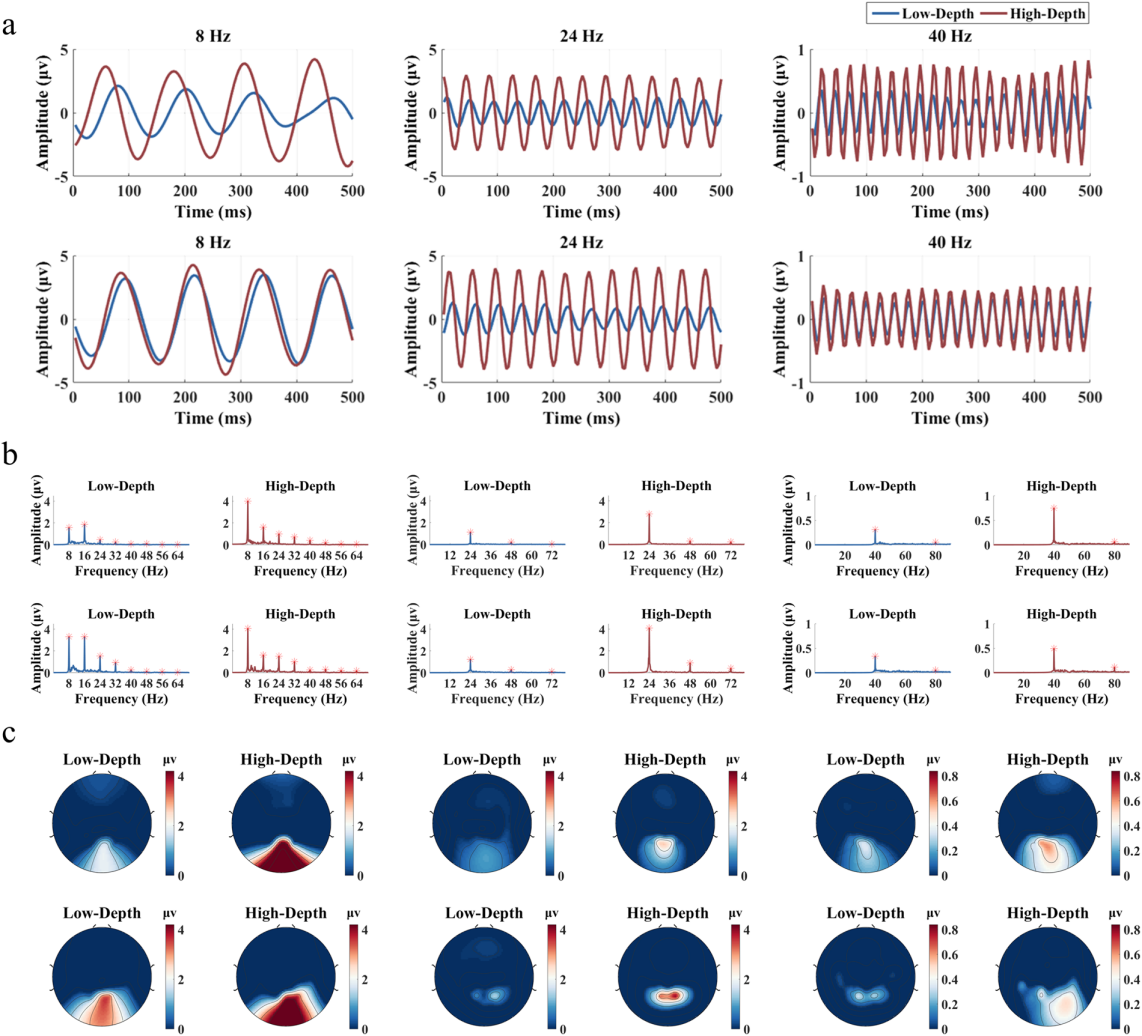
Stimulus signals and the corresponding SSVEP signals at 8 Hz, 24 Hz, and 40 Hz from two subjects. (**a**) Averaged SSVEP signals across 9 channels with a duration of 0.5 s. (**b**) Averaged SSVEP amplitude spectra across 9 channels. (**c**) Topographic maps on the amplitude of SSVEPs at the fundamental frequency for all channels on the scalp. The results in the first row of (**a**), (**b**) and (**c**) corresponds to the same participant, while the results in the second row corresponds to another participant. The filter parameters are consistent with those in Fig. 5.

To further investigate individual differences across different stimulation paradigms, Fig. 6 presents analyses of SSVEP characters from two distinct participants. Despite robust periodic responses at different stimulation frequencies (Fig. 6(b)), both participants exhibit distinct phase differences (Fig. 6(a)), possibly due to variations in the modulation depth of the two stimulation paradigms. In terms of spatial response analysis, under low frequency stimulations (e.g., 8 Hz), a significant portion of the occipital region is activated for both participants. However, under medium frequency stimulations, the second participant (the second row of Fig. 6(c)) activates a noticeably smaller region compared to the first participant (the first row of Fig. 6(c)). When exposed to high frequency visual stimulations, the second participant shows heightened sensitivity to the modulation depth of the stimulus compared to the first participant. These results underscore the significant individual difference within the dataset.

**Fig. 7:**
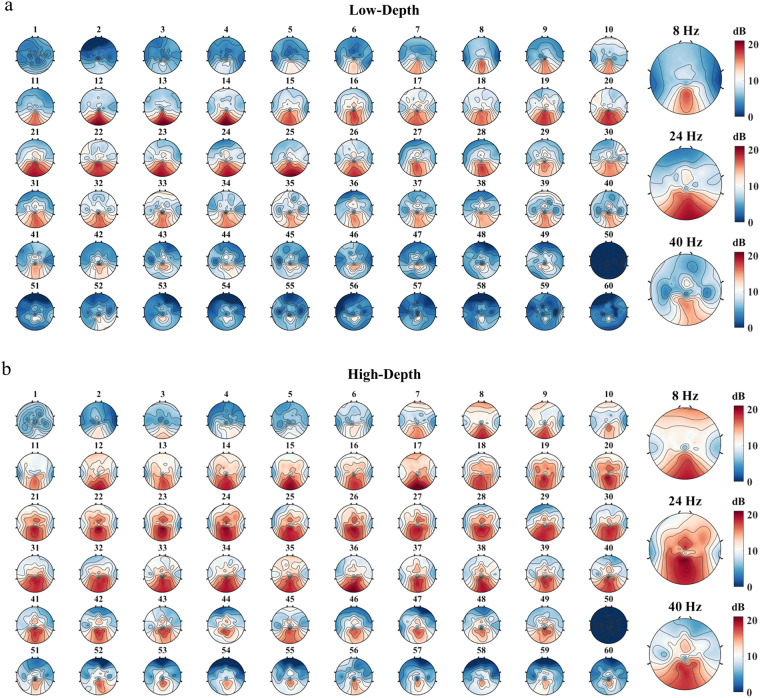
Averaged topographic map of SNR at 60 different stimulation frequencies for (**a**) low modulation depth stimulus and (**b**) high modulation depth stimulus across all participants. Enlarged images for 8 Hz, 24 Hz, and 40 Hz are on the right. The epoch data were all passed through a Chebyshev Type I band-pass filter with the range [f-0.2, f + 5] Hz, where f represents the stimulus frequency.

Figure 7 depicts the scalp response distribution of the averaged SNR across all participants as a function of stimulus frequency. Regions with high SNR and strong SSVEP response are mainly concentrated in the occipital area of the brain. With the increase in stimulus frequency, the size of the activation area first increases and then decreases. As shown in the enlarged figure on the right, the stimulus paradigm with a high modulation depth achieves a higher activation intensity. Across the two different modulation depth stimuli, the response at the fundamental frequency is strongest in the medium frequency band (e.g., 24 Hz), followed by either the low frequency band (e.g., 8 Hz) or the high frequency band (e.g., 40 Hz).

**Fig. 8:**
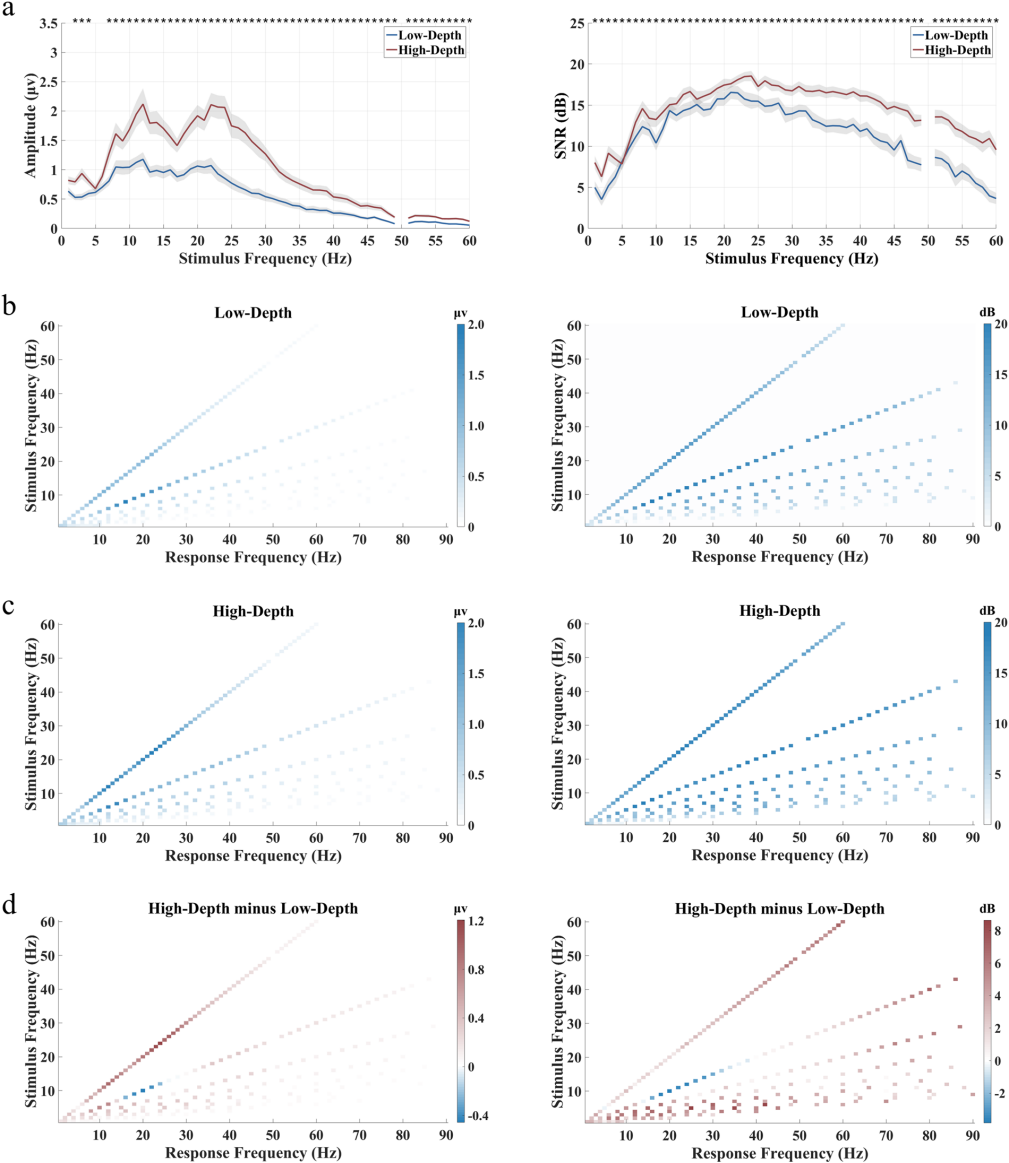
Averaged amplitude and SNR of SSVEPs for the two modulation depths across all participants. (**a**) The relationship between SSVEP amplitude and SNR with stimulation frequencies. The shaded area indicates the standard error. The asterisks indicate the significance level calculated by paired t-tests comparing the two stimulation paradigms at the same frequency (*p < 0.05). (**b**) The relationship between stimulus frequency and response frequency in the amplitude and SNR of SSVEPs for the low modulation depth paradigm. (**c**) The relationship between stimulus frequency and response frequency in the amplitude and SNR of SSVEPs for the high modulation depth paradigm. (**d**) The difference in the response amplitude and SNR between the two modulation depth conditions. The colour in (**b**), (**c**), and (**d**) represents the amplitude value or SNR at distinct response frequencies. The epoch data were all passed through a Chebyshev Type I band-pass filter with the range [0.8, 90] Hz.

The amplitude and SNR of SSVEPs at each stimulus frequency from 1 to 60 Hz under the two modulation depths are shown in Fig. 8(a). The stimulation with high modulation depth can evoke stronger SSVEP signals. Specifically, the two stimulation paradigms show significant differences in SNR across all frequencies from 1 to 60 Hz (p < 0.05). With the increase in stimulus frequency, the amplitude and SNR show a general trend of increasing and then decreasing. Amplitude response peaks occur at 12 Hz and 22 Hz, while the SNR reaches the highest around 20–25 Hz. Figure 8(b) and (c) illustrate the relationship between the stimulus frequency and response frequency in the spectrum for the two modulation depths, respectively. The frequency-following effect is evident at both the fundamental and harmonic frequencies, where the SSVEP response frequency linearly corresponds to the stimulus frequency across all harmonics. The SNR response at the harmonic peaks reaches up to 80 Hz. The difference between the Low-Depth and High-Depth conditions in Fig. 8(d) reveals that the high luminance stimuli induce a stronger fundamental response. However, the SNR of the second harmonic at certain stimulation frequencies (8–18 Hz) is inversely related to the stimulus luminance.

### Subjective evaluation analysis

**Fig. 9:**
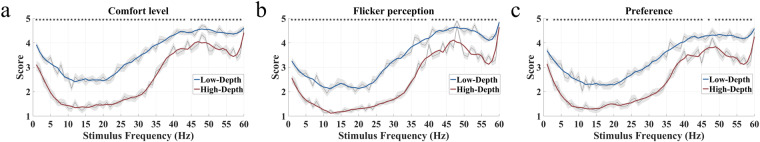
Mean subjective evaluation of (**a**) comfort level, (**b**) flicker perception, and (**c**) preference level for all stimulation frequencies. The shaded area indicates the standard error. The asterisks indicate the significance level calculated by paired t-tests comparing the two stimulation paradigms at the same frequency (*p < 0.05). The dashed line represents the real data, and the solid line is the result of using Savitzky-Golay filtering on the dashed line.

The average subjective evaluation results of each stimulus pattern across 30 subjects are shown in Fig. 9. The stimulus paradigm with a low modulation depth achieves a better user experience in terms of comfort level, flicker perception, and degree of preference. As the stimulus frequency increases, the user experience first deteriorates and then improves and tends to stabilize after 40 Hz. Among them, the user experience of the medium frequency band stimulation (10–20 Hz) is the worst. Although the ultra-low frequency band stimulation (1 Hz as an example) gives users a stronger flicker perception than the high frequency band stimulation (35 Hz as an example), they have a comparable level of comfort and preference. It is clearly shown that the user experience at 60 Hz is the best across the entire frequency band.

### Simulated BCI performance

The time-locked and phase-locked characteristics between the stimulus signal and the response EEG signals allow SSVEP to encode different targets with phase encoding^37^, frequency encoding^38^, or hybrid encoding^39^. This study simulated two types of four-target BCI systems by frequency coding and phase coding methods, respectively. Simulated classification validation on the dataset provides valuable insights for the design of SSVEP-BCI applications. The classification performance of SSVEP and user experience under different frequencies and modulation depths were quantitatively compared with the dataset.

### Frequency coding

For frequency coding, four frequencies were selected in each group of low, medium, and high frequency bands for classification (low: 2–5 Hz, medium: 12–15 Hz, high: 40–43 Hz). With frequency coding, the four-target BCI system for each frequency band was simulated offline, and the filter bank canonical correlation analysis^40^ (FBCCA) algorithm was used to calculate the classification accuracy. CCA^22^ is a commonly used algorithm for classifying SSVEP signals without training data, and FBCCA further improves CCA by combining SSVEP components at the fundamental and harmonic frequencies to detect SSVEPs. The advantage of the training-free algorithm lies in its ability to operate without prior model training, making it easy to use in practical applications. In this study, the harmonic number *N*_*h*_ for the sine-cosine template was set to 10, and the parameters of the filter bank (weight vectors *ω* and the number of sub-bands *N*) were optimized by a grid search method towards the highest classification accuracy. The weight vector *ω* is calculated as follows:^40^

6 $$\begin{array}{c}\omega (n)={n}^{-a}+b,n\in [1,N]\end{array}$$

where *a*, *b*, and *N* are limited to [0:0.25:2], [0:0.25:1], and [1:1:10], respectively. The optimal filter bank parameters obtained by the grid search method were listed as follows: Low-Depth: *a* = 0, *b* = 0, *n* = 10; High-Depth: *a* = 0, *b* = 0, *n* = 10 (in the low frequency band); Low-Depth: *a* = 0, *b* = 0, *n* = 2; High-Depth: *a* = 0, *b* = 0, *n* = 3 (in the medium frequency band); Low-Depth: *a* = 0, *b* = 0, *n* = 1; High-Depth: *a* = 0, *b* = 0, *n*=1(in the high frequency band).

**Fig. 10:**
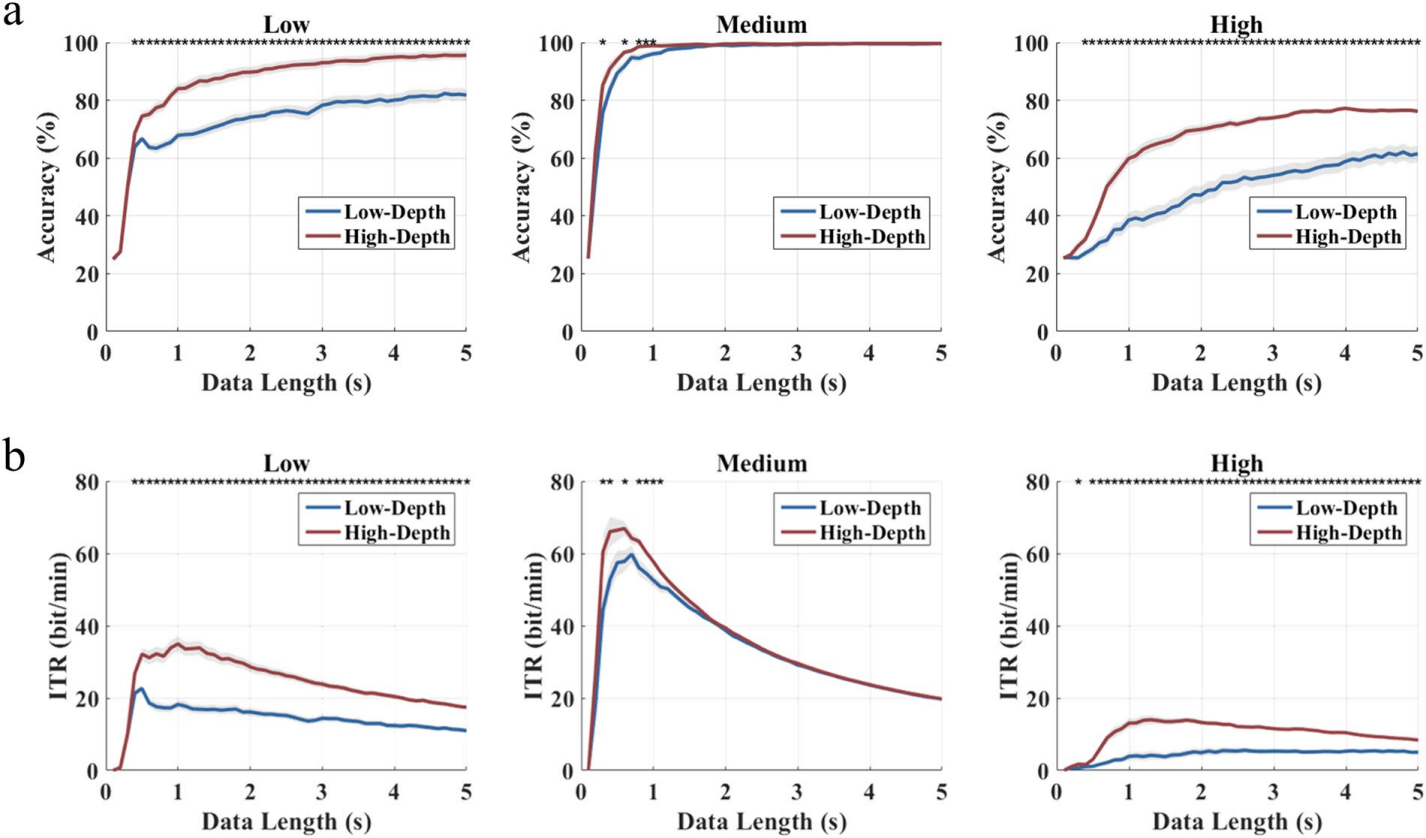
Averaged (**a**) classification accuracy and (**b**) ITR for low, medium, and high frequency bands with different data lengths. The asterisks indicate the significance level calculated by paired t-tests comparing the two stimulation paradigms at the same frequency (*p < 0.05). The shaded area indicates the standard error. The epoch data were all passed through a Chebyshev Type I band-pass filter with the range [m, 90] Hz, where the values of parameter “m” for the three frequency bands were set to 1.8, 5.8, and 32.8 (Low-Depth); 1.8, 8.8, and 35.8 (High-Depth).

Classification accuracy and ITR at different data lengths from 0.1 s to 5 s (interval 0.1 s) are shown in Fig. 10. For the low frequency band, the average classification accuracy across all subjects increases rapidly with the increase of data length before 0.5 s and then increases slowly to 5 s for both modulation depths (0.1 s: 24.93%/25.07%, 0.5 s: 66.74%/74.51%, 5 s: 81.81%/95.56%). For the medium frequency band, the classification accuracy of the High-Depth condition tends to saturate earlier than that of the Low-Depth condition (High-Depth: 98.54% at 0.8 s; Low-Depth: 97.50% at 1.2 s). The ITR reached its highest point in less than 1 s (Low-Depth: 59.83 bits/min at 0.7 s; High-Depth: 66.93 bits/min at 0.6 s). For the high frequency band, the classification accuracy increases with longer data length, like the low frequency band. The classification accuracy of the high frequency band is the lowest among the three bands, and the performance of the medium frequency band is the best. In addition, in both low frequency and high frequency bands, the High-Depth condition achieves significantly better classification performance than the Low-Depth condition.

**Fig. 11:**
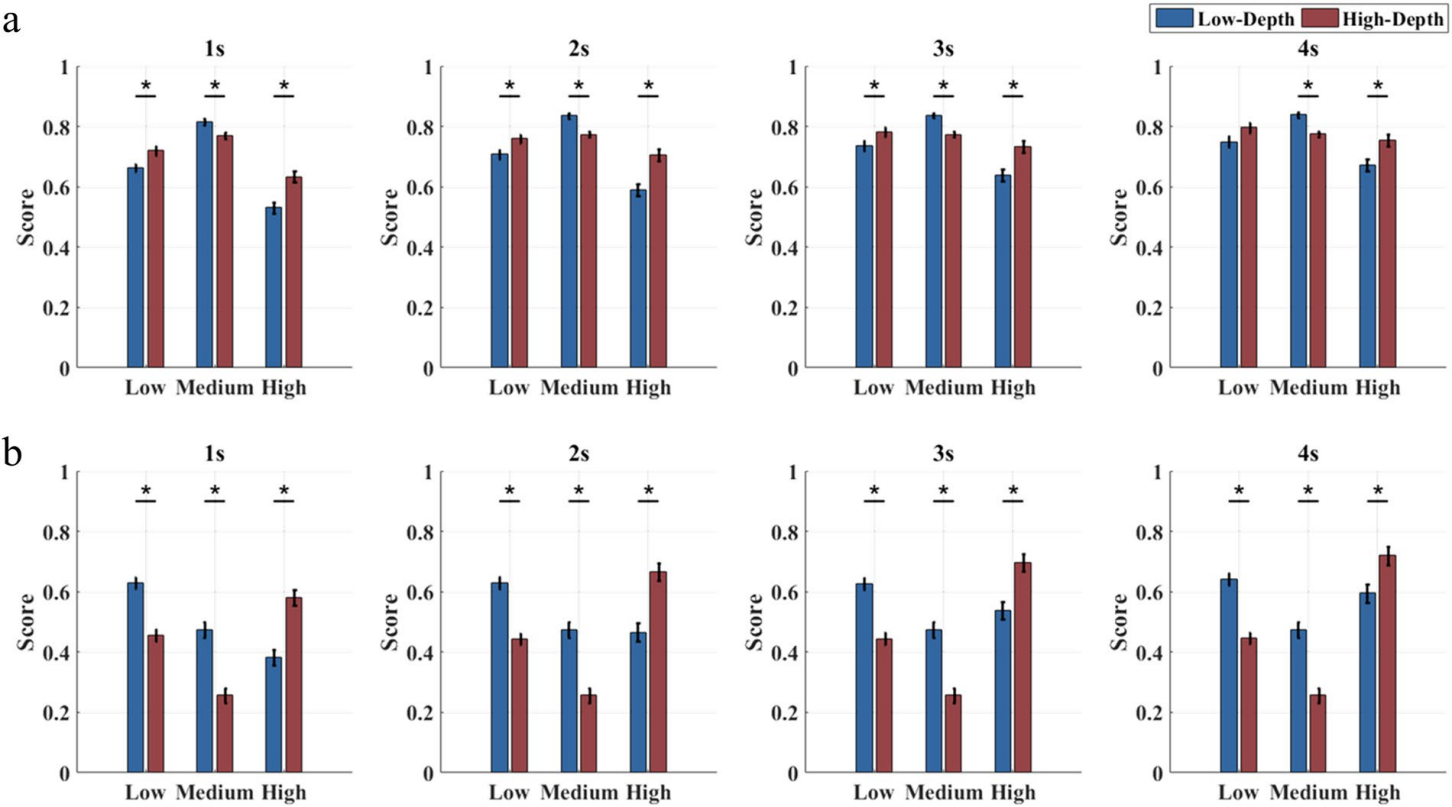
Composite scores that weight classification performance and user experience at different data lengths for the frequency coding BCI paradigms. (**a**) The weight ratio of classification accuracy to user experience is 7:3. (**b**) When the classification accuracy is less than 70%, the weight ratio of classification accuracy to user experience is 1:0, otherwise, the weight ratio is 0:1. The asterisks indicate the significance level calculated by paired t-tests comparing the two stimulation paradigms (*p < 0.05).

When designing a BCI system for practical applications, it is necessary to jointly consider system performance and user experience^41^. Therefore, this study proposes to weight classification accuracy and user experience to choose the optimal stimulus frequency band and modulation depth. The subsequent analysis involved a validation conducted with two illustrative weighting methods. Figure 11(a) shows the combined score at different data lengths, which is calculated using a 7:3 ratio between normalized classification accuracy and subjective score. At all data lengths, the composite score for the medium frequency band stimuli is the highest due to the highest classification accuracy among the three frequency bands. As the length of the data increases, the difference between the three bands decreases, mainly due to the improvement of the accuracy of low and high frequency bands. For different data lengths (except for a data length of 4 s), the overall performance of the High-Depth condition is significantly better than that of the Low-Depth condition in both low and high-frequency bands (p < 0.05). However, the phenomenon is opposite in the medium frequency band due to the better user experience for the Low-Depth condition and similar classification accuracy between the two modulation depths. Figure 11(b) adopts a conditional weighting scheme. Specifically, if the classification accuracy is less than 70%, the weight ratio of classification accuracy to user experience is 1:0. Otherwise, the weight ratio is 0:1. For both the two modulation depths, the combined score of the medium frequency band is the lowest, because the subjective evaluation of this band is the worst. What’s more, the combined scores of the Low-Depth at the low and medium frequency bands are significantly higher than that of the High-Depth condition (p < 0.05), and the result is opposite at the high frequency band. The second weighting scheme presents a different result because it emphasizes user experience when the BCI system can normally work.

### Phase coding

For phase coding, data epochs extracted with different time shifts were used to evaluate classification performance for each stimulus frequency. According to the stimulus onset, the classification of four-class SSVEPs with initial phase values of 0°, 90°, 180°, and 270° was conducted for each stimulus frequency. Data for the first 140 ms of each trial were excluded from the analysis to avoid the interference of the transient event-related potential. The remaining data were divided into four 1-second-long SSVEP segments coded by four phases. For example, when the stimulus frequency is 25 Hz, there are 40 data points per cycle, therefore the time shift of the four different phases is 0 s, 0.01 s, 0.02 s, 0.03 s, and the corresponding number of time-shifted data points is 0, 10, 20, and 30 respectively. In this way, four-class SSVEP segments (12 trials for each class) with the same stimulus frequency but different phases can be obtained for each modulation depth of each stimulus frequency. The filter bank^40^ TRCA^15^ algorithm and the leave-one-out cross validation were used to estimate the offline classification accuracy. The supervised algorithm capable of learning from individual data can generate personalized templates, enhancing the ability to capture individual differences and variability. This approach contributes to improving the classification accuracy and overall performance of SSVEP-BCIs. The number of filter bank sub-bands and the weight vector parameters for each sub-band were also determined using the grid search method, and the scanning ranges were the same as those in the frequency coding method.

**Fig. 12:**
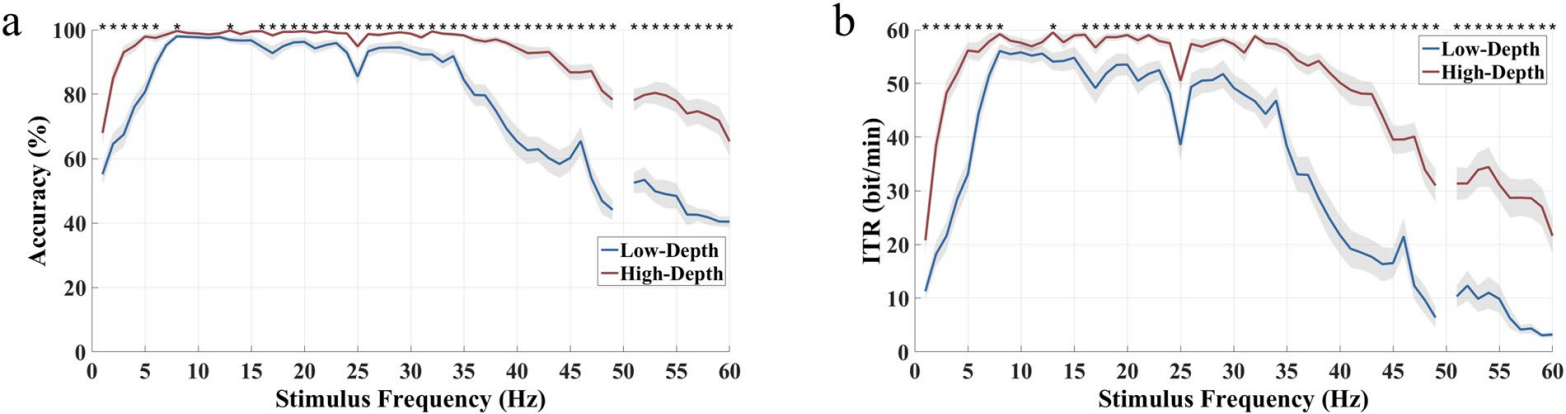
Averaged (**a**) classification accuracy and (**b**) ITR for each stimulus frequency of two modulation depths. The asterisks indicate the significance level calculated by paired t-tests comparing the two stimulation paradigms (*p < 0.05). The shaded area indicates the standard error. The epoch data were all passed through a Chebyshev Type I band-pass filter with the range [m, 90] Hz, where the values of parameter “m” were determined towards the highest classification performance.

The accuracy and ITR of offline classification at a data length of 1 s are shown in Fig. 12. At most frequencies, a significant difference in accuracy and ITR between two modulation depths was obtained. In the range of 1–60 Hz, with the increase of stimulus frequency, both accuracy and ITR curves show a trend of first rising to saturation and then decreasing, and the accuracy of the High-Depth stimulation is higher than that of the Low-Depth stimulation. In the range of 9–15 Hz (except for 13 Hz), the difference between the two curves is not significant (p > 0.05), where the offline accuracy reaches more than 95% and the corresponding ITR value exceeds 49 bits/min. After 30 Hz, the accuracy of the Low-Depth stimulation decreases sharply, with a difference between the two modulation depths up to 34% (81.11% vs 46.88% at 48 Hz), and the High-Depth stimulation shows a slower decline. The High-Depth stimulations have an accuracy rate of more than 65% across the entire frequency band, while the Low-Depth stimulations only maintain this level in the 3–40 Hz band.

**Fig. 13:**
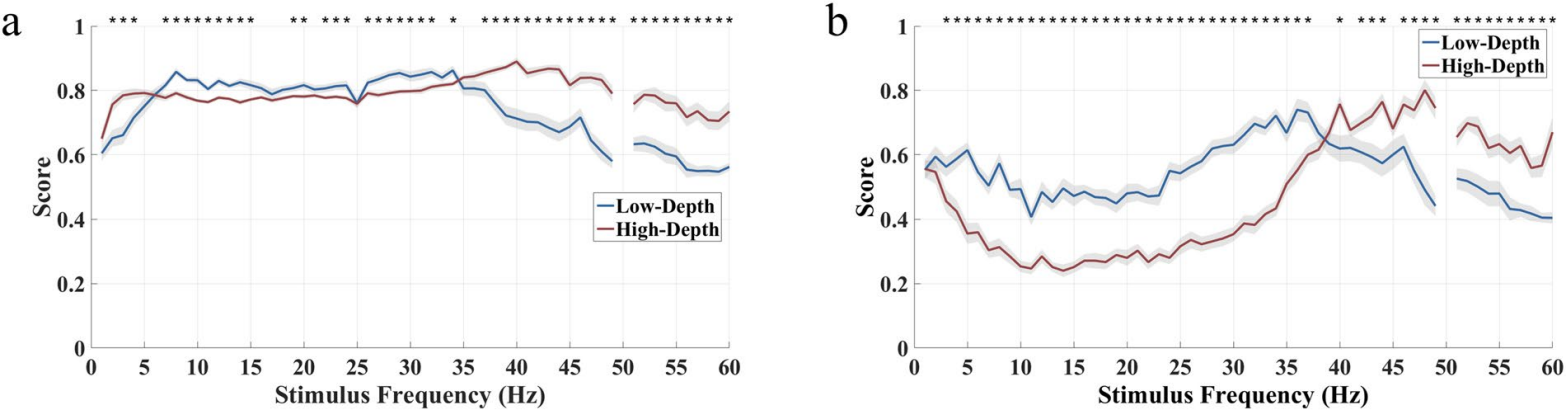
Composite scores that weight classification performance and user experience for the phase coding BCI paradigms. (**a**) The weight ratio of classification accuracy to user experience is 7:3. (**b**) When the classification accuracy is less than 70%, the weight ratio of classification accuracy to user experience is 1:0. Otherwise, the weight ratio is 0:1.

Furthermore, this study assessed the overall performance by incorporating a blend of classification accuracy and subjective scores in the weighting scheme. Figure 13 shows the results of using the same weighting scheme for frequency coding. When the ratio between classification performance and user experience is 7:3, the Low-Depth stimulus in the 8–34 Hz frequency band has higher scores than the High-Depth stimulus. However, it should be noted that the paired t-tests show that there is a significant difference in the comprehensive score of stimulation between the two modulation depths at most stimulus frequencies. In the ultra-low frequency (<6 Hz) and high frequency band (>35 Hz), the High-Depth stimulations show higher scores. As shown in Fig. 12(b), when adopting a conditional weighting scheme, the comprehensive scores show that the Low-Depth stimulus in the 1–38 Hz band is preferable. The difference between the two weighting schemes is mainly reflected in the ultra-low frequency band (<6 Hz).

## Usage Notes

We provide continuous EEG recordings and data epochs time-locked to the stimuli in this study. For routine offline analyses, such as screening frequency subsets when designing a BCI system or the topology analysis of the brain associated with visual stimuli, data epochs can be downloaded for free for analysis.

## Data Availability

Custom codes for processing the data and the figures can be obtained for free from Figshare^42^ (10.6084/m9.figshare.23641092). The data processing and technical validations were conducted in MATLAB R2015b. A “README.txt” file was used for a brief description of the code in the code repository.
